# Myostatin mediates abdominal aortic atherosclerosis progression by inducing vascular smooth muscle cell dysfunction and monocyte recruitment

**DOI:** 10.1038/srep46362

**Published:** 2017-04-13

**Authors:** D. Verzola, S. Milanesi, M. Bertolotto, S. Garibaldi, B. Villaggio, C. Brunelli, M. Balbi, P. Ameri, F. Montecucco, D. Palombo, G. Ghigliotti, G. Garibotto, J. H. Lindeman, C. Barisione

**Affiliations:** 1 Nephrology Division, Department of Internal Medicine, IRCCS University Hospital San Martino, University of Genova, Genova, Italy; 2 First Clinic of Internal Medicine, Department of Internal Medicine, University of Genova, viale Benedetto XV, 6, 16132 Genova, Italy; 3 Division of Cardiology, IRCCS University Hospital San Martino, Research Centre of Cardiovascular Biology, University of Genova, Genova, Italy; 4 IRCCS AOU San Martino-IST, Genova, largo Benzi 10 16143 Genova, Italy; 5 Unit of Vascular and Endovascular Surgery, University of Genova, Genova, Italy; 6 Department of Vascular Surgery, Leiden University Medical Center, Leiden, The Netherlands

## Abstract

Myostatin (Mstn) is a skeletal muscle growth inhibitor involved in metabolic disorders and heart fibrosis. In this study we sought to verify whether Mstn is also operative in atherosclerosis of abdominal aorta. In human specimens, Mstn expression was almost absent in normal vessels, became detectable in the media of non-progressive lesions and increased with the severity of the damage. In progressive atherosclerotic lesions, Mstn was present in the media, neointima, plaque shoulder and in infiltrating macrophages. Mstn co-localized with α-smooth muscle actin (α-SMA) staining and with some CD45+ cells, indicating Mstn expression in VSMCs and bloodstream-derived leukocytes. *In vitro*, Mstn was tested in VSMCs and monocytes. In A7r5 VSMCs, Mstn downregulated proliferation and Smoothelin mRNA, induced cytoskeletal rearrangement, increased migratory rate and MCP-1/CCR2 expression. In monocytes (THP-1 cells and human monocytes), Mstn acted as a chemoattractant and increased the MCP-1-dependent chemotaxis, F-actin, α-SMA, MCP-1 and CCR2 expression; in turn, MCP-1 increased Mstn mRNA. Mstn induced JNK phosphorylation both in VSMCs and monocytes. Our results indicate that Mstn is overexpressed in abdominal aortic wall deterioration, affects VSMCs and monocyte biology and sustains a chronic inflammatory milieu. These findings propose to consider Mstn as a new playmaker in atherosclerosis progression.

The atherosclerotic process is a chronic inflammatory condition that occurs principally in large and medium-sized elastic and muscular arteries. Its development is sustained by a multifactorial ensemble of cues that acts synergistically. Plaque growth can remain clinically silent for years, and can suddenly trigger major clinical events, i.e. stroke, myocardial infarction, abdominal aortic aneurysm rupture, depending on the affected tracts of the artery tree. Indeed, atherosclerotic lesions develop in lesion-prone areas of the vascular bed with different speed, depending on systemic levels and local actions of bio-humoral cues, such as oxidized LDL, and on disturbed haemodynamic forces impacting on endothelial lining and on the vessel wall, as occurs in untreated arterial hypertension. In humans, the abdominal aorta represents a district where atherogenesis occurs; overtime, progression of vessel wall damage leads to loss of aortic elasticity and deterioration of the vessel wall. This may result in the development of life threatening complications as the fissure and the rupture of an abdominal aortic aneurysm (AAA), peripheral embolization and critical limb ischemia^1^,^2^.

Major hallmarks of the atherosclerotic process include vascular smooth muscle cell (VSMC) activation, monocyte-macrophage recruitment and their reciprocal interplay in vessel wall areas predisposed to atherosclerotic lesions development^3^,^4^,^5^,^6^. VSMCs change their phenotype in response to local stimuli such as mechanical stretch, growth factors or cytokines, displaying a wide variety of features. At large, it is assumed that VSMCs can switch from a well-defined contractile, steady-state status back to a synthetic, proliferative phenotype^7^,^8^. However a local co-expression of characteristic protein markers of contractile (Smoothelin, SM22 alpha) and secretory (Collagen 1) phenotypes has been observed in VSMCs from Marfan patients and has been linked to the dysregulation of the transforming growth factor (TGF)-β system^9^. These findings reveal how the modulation of the VSMC phenotype is a complex, multifaceted process resulting from the interaction of several cellular pathways and extracellular signals. Besides their structural functions, VSMCs can also drive vascular inflammation as demonstrated by the up-regulation of CCR2 and MCP-1 expression in response to biochemical stimuli and during arterial aging^10^,^11^.

MCP-1 expression, one of the main mediators of vascular inflammation, is triggered by the activation of several transcriptional factors and of different signaling pathways, including MAPkinases, depending on the involved cell type^12^,^13^,^14^.

Myostatin (Mstn), also known as growth differentiation factor 8 (GDF-8), is a member of the TGF-β super family, which was first described in skeletal muscle and characterized as a negative regulator of skeletal muscle growth^15^. It has since then been found in other tissues in both mammalian and nonmammalian systems^16^. Mstn has been proposed as a putative new mediator of heart dysfunction, cardiac and extra-cardiac tissue fibrosis and cardiovascular cachexia^17^,^18^,^19^,^20^,^21^.

Besides causing cachexia, recent observations suggest that Mstn might also play a role in vascular damage directly or interacting with Activin (with which it shares receptors) or TGF-β signaling. Mstn and its receptor Activin type II-r are both abundantly expressed in mouse aorta^22^,^23^ and genetic inactivation of Mstn in Ldlr null mice blunts diet-induced atherosclerosis^24^. Moreover, the exposure of aortic endothelial cells to Mstn leads to activation of TGF-β signaling, decrease of the endothelial NO synthase (eNOS) phosphorylation and increased expression of pro-atherogenic adhesion molecules ICAM-1 and VCAM-1^23^. These findings suggest that the components of vessel wall are direct targets of Mstn, and that therefore Mstn may be relevant for diet-induced metabolic disorders^25^.

Central in our study has been to detect Mstn in aorta specimens and to define whether Mstn tissue levels change in association to the progression of abdominal aortic atherosclerosis. To this end, we first evaluated Mstn expression and localization in a collection of human peri-renal aortic specimens that comprises the full spectrum of atherosclerotic stages. We then investigated *in vitro* the mechanisms by which Mstn might affect VSMC and monocyte biology. We observed that Mstn, mainly localized in resident medial VSMCs during early aortic wall remodeling, is also found in the neointima, neovessels and in infiltrating cells at site of atherosclerotic lesions with the progression of the aortic vascular damage. Our *in vitro* observations proved that Mstn induces vascular inflammatory changes by upregulating MCP-1 and CCR2 axis both in VSMCs and monocytes and indicate JNK phosphorylation as the common intracellular signaling elicited in both cell types. Taken together, these results suggest a substantial role for Mstn in the process of atherosclerosis development.

## Results

### Mstn expression in human atherosclerotic lesions

Mstn expression was evaluated by immunohistochemistry in human peri-renal aortic specimens including normal aortae, aortae with non-progressive intimal lesions and aortae with progressive atherosclerotic lesions, up to the stage of fibrotic calcified plaques (FCP), as referred by Van Dijk *et al*.^26^.

**Figure 1:**
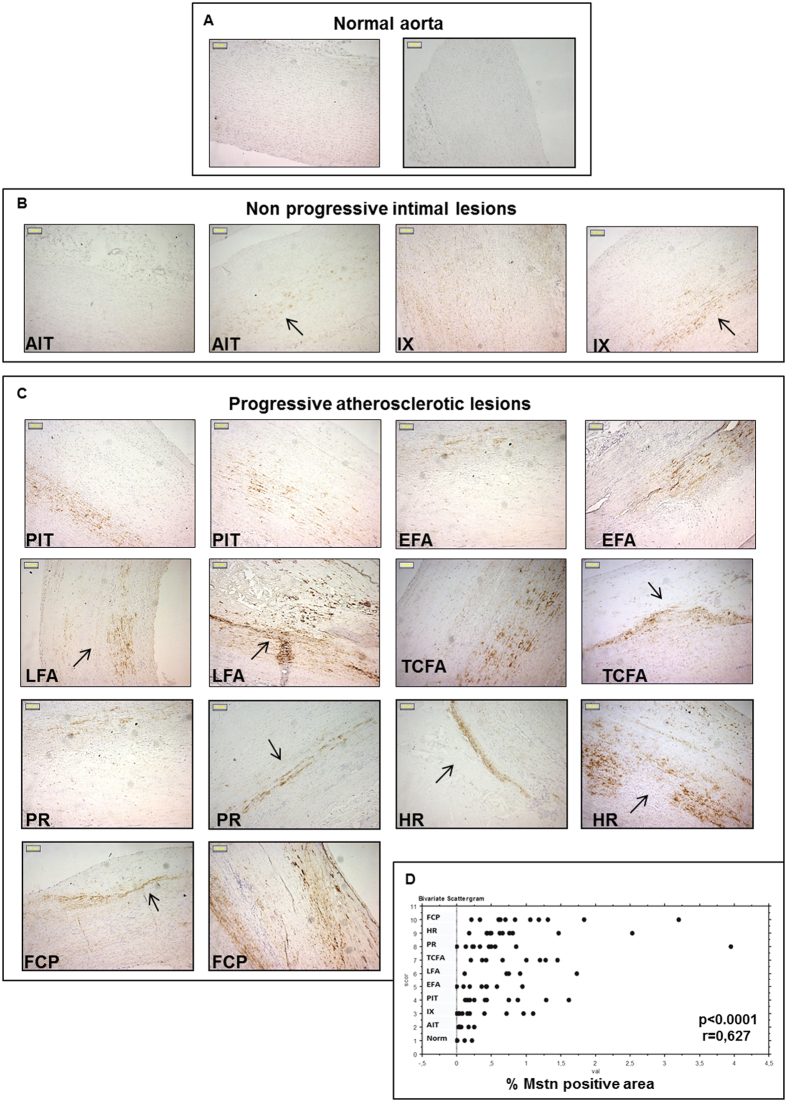
Expression of Myostatin (Mstn) in human peri-renal aortic specimens evaluated by immunohistochemistry. Representative pictures of normal aortas (**A**), non-progressive intimal lesions (**B**) and progressive atherosclerotic lesions (**C**). (Magnification: x100; Bars = 50 μM). The arrows indicate positive areas; (**D**) Image analysis of Mstn immunopositivity plotted in relation to the type of atherosclerosis (Norm: Normal aorta; AIT: adaptive intimal thickening; IX: Intimal xanthoma; PIT: pathological intimal thickening; EFA: Early fibroatheroma; LFA: late fibroatheroma; TCFA: thin cap fibroatheroma; PR: plaque rupture; HR: healed plaque rupture; FCP: fibrotic calcified plaque). Mstn expression significantly correlates with lesion type progression (R = 0.627, p = 0.0001).

Mstn was either absent or minimally expressed in normal aorta (Fig. 1A), while it was already detectable in non-progressive lesions (Fig. 1B). In adaptive intimal thickening (AIT), it was distributed uniformly and faintly localized in the inner media layer. In intimal xanthoma (IX) Mstn displayed a variable distribution with a pale signal or a strong expression in both media and intima and at the edge of the xanthoma.

In progressive atherosclerotic lesions (Fig. 1C), an increased, patchy Mstn positivity was observed. In pathological intimal thickening (PIT) and early fibroatheroma (EFA), it diffused from the media to the intimal-medial border zone. In late fibroatheroma (LFA), Mstn attributable signal was more pronounced and involved media, neointima and fibrotic cap.

At later stages, when thinning of fibrotic cap (thin cap fibroatheroma, TCFA, and plaque rupture, PR) occurred, Mstn accumulated in the media along the border and within the cellular component of the plaques. The pattern of Mstn expression was similar even in the most advanced lesions, suggesting that Mstn is present already in early stages of vessel-wall damage and participates to the propagation of the wall injury in a media-intima direction. Quantification by image analysis revealed a rapid increase of the Mstn immunohistochemical signal from normal aortas to aortas with atherosclerotic lesions, with a strong positive correlation between the Mstn expression and the severity of vascular damage (r = 0.627, p < 0.0001) (Fig. 1D); the presence of Mstn within atherosclerotic lesion was also verified by Western Blot, as shown in Supplementary Figure S1.

**Figure 2:**
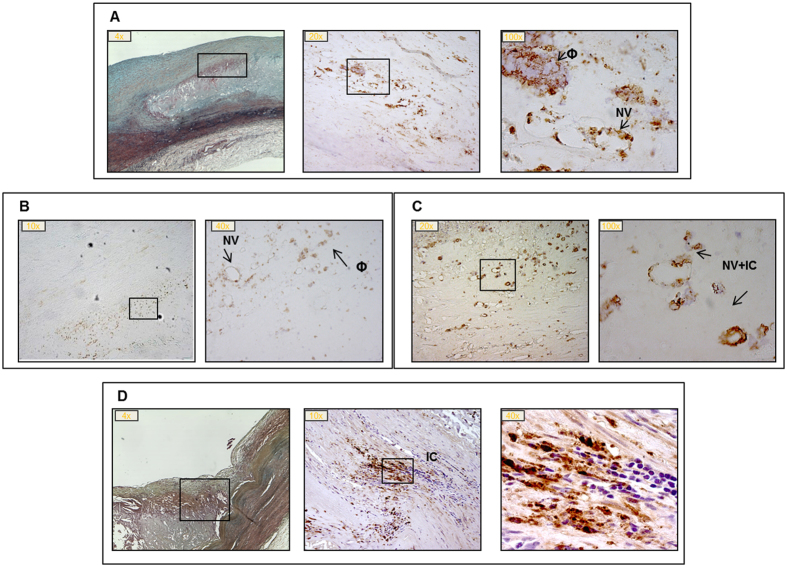
Myostatin (Mstn) immunostaining in progressive atherosclerotic lesions. Panel A: late fibroatheroma, LFA; panel B: thin cap fibroatheroma, TCFA; panel C: plaque rupture, PR; panel D: healed plaque rupture, HR. Boxes indicate segments with Mstn positivity in correspondence of neovasa (NV), macrophages (Φ) and infiltrating cells (IC); magnification: 4-100x. Panel A and D: the first left image is stained with Movat pentachrome.

Within the lesions, starting from LFA, Mstn immunopositivity was found in infiltrating macrophages in correspondence of the plaque shoulders and in proximity of neovasa (Fig. 2A). Also, we observed a more consistent presence of Mstn+ infiltrating cells (Fig. 2B–D).

The observed semi-quantitative changes of Mstn immunostaining in the different areas of interest are summarized in Table 1.

As a next step, we studied Mstn gene expression in specimens bearing early and advanced atherosclerotic lesions (Supplementary Figure S2); Vimentin was used as housekeeping, due to the preliminary observation of a predominant Mstn signal in VSMCs of the tunica media. We found no significant difference in Mstn gene expression between the two groups considered, but we observed a wide discrepancy among samples belonging to the same lesion type. Our explanation for this finding is that truly increased Mstn signal in VSMCs occurs early during vascular remodeling and may retain a spot-like distribution.

### Mstn localization in resident VSMCs and bloodstream derived cells

**Figure 3:**
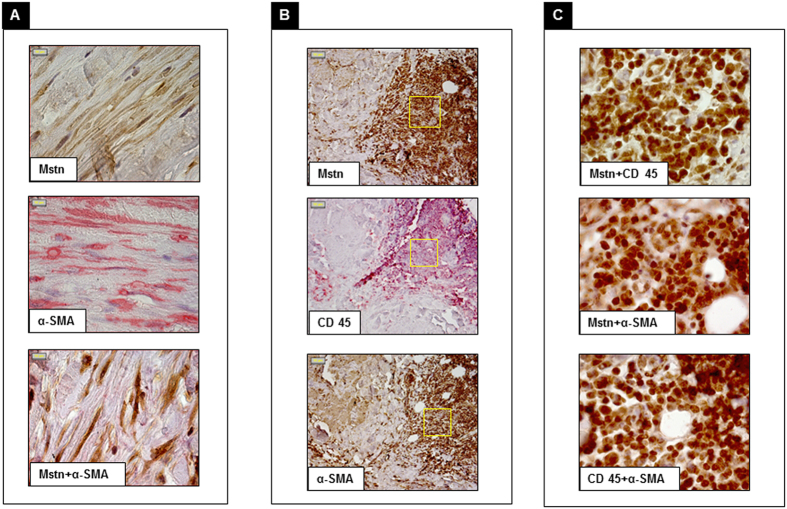
Immunohistochemical double staining of aortic cellular components associated with Myostatin (Mstn). Panel A: Mstn (brown) and α smooth muscle actin (α-SMA) (red); merged image shows that Mstn colocalizes with α-SMA. Panel B: staining with Mstn (brown), CD45 (red) and α-SMA (brown). Panel C: merged images of Mstn and CD45, Mstn and α-SMA (Mstn brown; CD45 and α-SMA red) and CD45 and α-SMA (brown and red respectively), Mstn colocalizes with CD45 and α-SMA and the latter with CD45; fields correspond to boxes in panel B (Magnification: 100x panel A,C; 20x panel B).

To gain information on the cell types responsible for Mstn enrichment within the vessel wall, we first performed colocalization studies of Mstn with α-smooth muscle actin (α-SMA) and CD45, markers of VSMCs and of cells from haematopoietic lineage respectively. Mstn staining co-localized with that for α-SMA (Fig. 3A) and with patches of CD45+ cells. Interestingly, some CD45+/Mstn+ cells were also positive for α-SMA, considered as a surrogate marker of fibrosis, leading to hypothesize the commitment of bloodstream derived cells toward a fibroblast-like differentiation within the vessel wall (Fig. 3B,C).

### Effects of Mstn on VSMCs activation

Mstn is a secreted signaling molecule with an inhibitory effect on cell growth and differentiation whose effects on skeletal muscle cells are mainly exerted in an autocrine-paracrine manner^27^.

To address the question whether local amount of Mstn in the vessel wall induces medial VSMCs activation, we tested *in vitro* the effect of Mstn treatment (500 ng/ml) in cultured rat aortic VSMCs (A7r5) and the following parameters were evaluated:

#### Cell cycle, proliferation and viability

**Figure 4:**
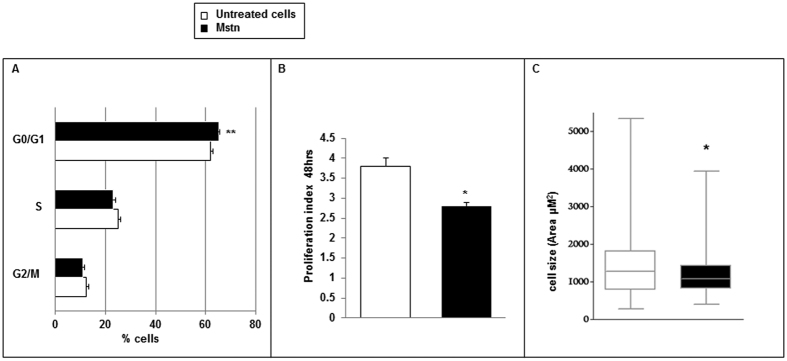
Effects of Myostatin (Mstn) on A7r5 cell cycle (**A**), proliferation index (**B**) and cell size (**C**). A 24 hour Mstn treatment caused an increase of G0/G1 phase as revealed by propidium iodide staining and flow-cytometry reading. At 48 hours theproliferation rate as revealed by CFSE-DA assay and the cell size were reduced. Data shown as mean ± SEM (**A,B**) and median ± IQR (**C**). *p < 0.05, **p < 0.01 vs. untreated cells (n = 3).

A 24 hour Mstn exposure lowered the cell cycle progression by increasing the G0/G1 phase (Mstn treated cells 65 ± 0.1%; untreated cells 62.6 ± 0.1%; p < 0.01), thus reducing the proliferation rate, expressed as Proliferation Index (PI), after 48 hours (Mstn treated cells 2.8 ± 0.11; untreated cells 3.8 ± 0.2; p < 0.05) (Fig. 4A,B).

After Mstn supplementation, we also observed a restriction in the range of cell size distribution as indicated by the surface area, with an overall reduction of cell dimension (Mstn treated cells: 1072 μM^2^ [820–1433 μM^2^], untreated cells 1281 μM^2^ [777–1778 μM^2^], median [interquartile range]; p < 0.05) (Fig. 4C); this finding is not attributable to a change in the apoptotic rate (data not shown).

#### Cytoskeletal structure and migratory rate

**Figure 5:**
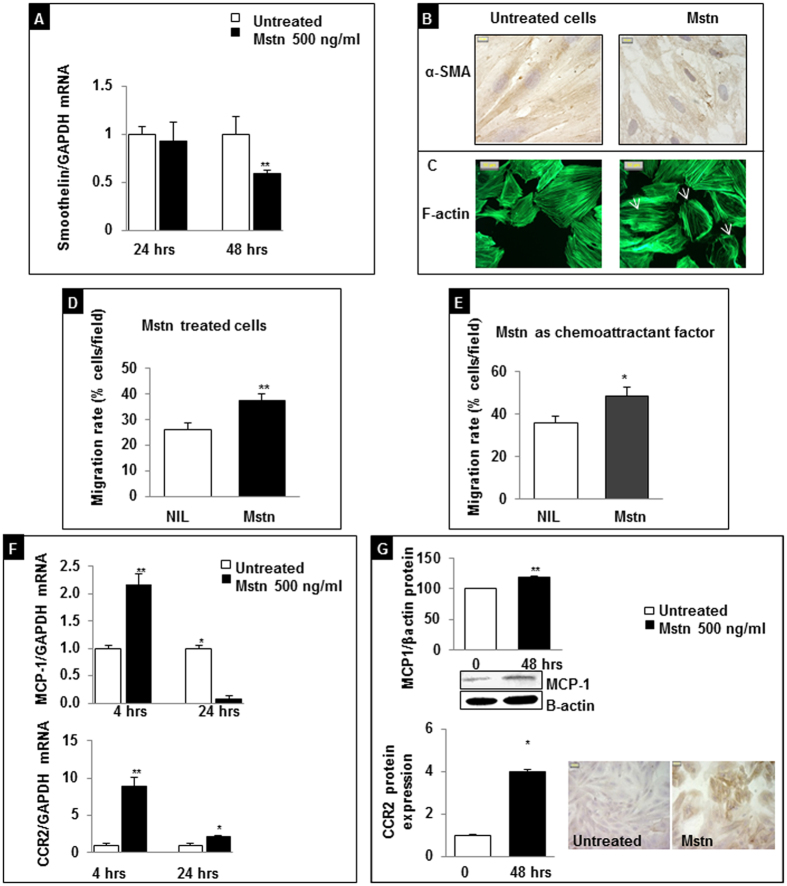
Effects of Myostatin (Mstn) on VSMC phenotype. (**A**) Smoothelin mRNA expression analyzed by real-time RT-PCR. All data were first normalized to GAPDH mRNA and values were expressed as fold increase ± SEM versus untreated cells. **p = 0.0054. (**B**) Evaluation of cytoskeleton organization by immunostaining: α smooth muscle actin (α-SMA) network in Mstn treated cells is deranged respect to control cells (magnification 100x). (**C**) F-actin stress fiber organization visualized with phalloidin-FITC in the absence or presence of Mstn. A 24 hour Mstn treatment affected F-actin organization. Arrows indicate cortical F-actin distribution (magnification 40x). (**D**) Mstn induces VSMC migration. **p < 0.01 vs. untreated cells. (**E**) Mstn acts as a chemoattractant stimulus on VSMCs. *p < 0.05 vs. untreated cells. (F) Effects of Mstn on MCP-1 and CCR2 mRNA levels determined by real time PCR after 4 and 24 hour exposition. Values are expressed as fold increase ± SEM versus untreated cells. *p < 0.05, **p < 0.01. (**G**) MCP-1 and CCR2 protein expression as evaluated by western blot and immunocytochemistry respectively, after a 48 hour treatment.

To this end, we evaluated the gene expression of Smoothelin, a structural protein that promotes contractile phenotype and myogenic tone exclusively in VSMCs^28^,^29^. After a 24 hour exposure to Mstn, Smoothelin expression was unchanged compared to untreated A7r5 cells, while it was significantly reduced (about 40%) after a 48 hour exposure (p < 0.01) (Fig. 5A).

Smoothelin associates with stress fibers by binding to α-SMA and polymerized actin and constitutes part of the cytoskeleton. To test whether Mstn affects also other elements of the cytoskeletal apparatus, we stained with α-SMA antibody after a 24 hour of Mstn exposure; we observed a decreased positivity and a pattern reflecting derangement of its architecture, with loss of the stress fiber alignment and disorganized orientation (Fig. 5B).

Then, we studied F-actin intracellular distribution: the AlexaFluor 488-conjugate-phalloidin staining revealed a compact polymerization of F-actin in stress fibers along the major cell axis in untreated cells, while Mstn treatment lead to its re-arrangement in thinner and poorly oriented fibers localized at cortical level (Fig. 5C).

As a consequence Mstn promoted an increased migration of VSMCs: indeed, a 16 hour pre-incubation with 500 ng/ml Mstn increased the chemotactic index of A7r5 cells toward a complete medium (37.5 ± 2.6% vs. 26.9 ± 2.5%, Mstn vs. untreated cells, p = 0.007) (Fig. 5D); when added to the lower well of the Boyden chamber, Mstn acted as a potent chemoattractant factor (48 ± 4.2% vs. 36 ± 3.1%, Mstn vs. untreated cells, p = 0.03) (Fig. 5E).

#### Expression of MCP-1 and its receptor CCR2

Mstn treatment early induced a strong upregulation of MCP-1 and CCR2 expression mRNA (2.2 ± 0.26 folds and 8.9 ± 1.2 folds versus control, respectively, after 4 hour treatment; p < 0.01) (Fig. 5F). This response is mirrored at protein level for longer times of exposure, leading to a 20% increase of MCP-1 protein expression as evaluated by western blot and a four-fold increased immunopositivity for CCR2, as detected by immunocytochemistry at 48 hours (Fig. 5G), suggesting that in VSMCs Mstn induces proinflammatory changes previously observed in response to biochemical stimuli and during arterial aging^10^.

### Effects of Mstn on monocyte activation

To define the biological relevance of Mstn expression in CD45+/Mstn+ cells in atherosclerotic lesions, we then designed experiments where a monocyte cell line (THP-1 cells) and human freshly isolated monocytes were exposed to Mstn (500 ng/ml) or to MCP-1 (10 ng/ml).

#### α-SMA, MCP-1 and CCR2 expression

**Figure 6:**
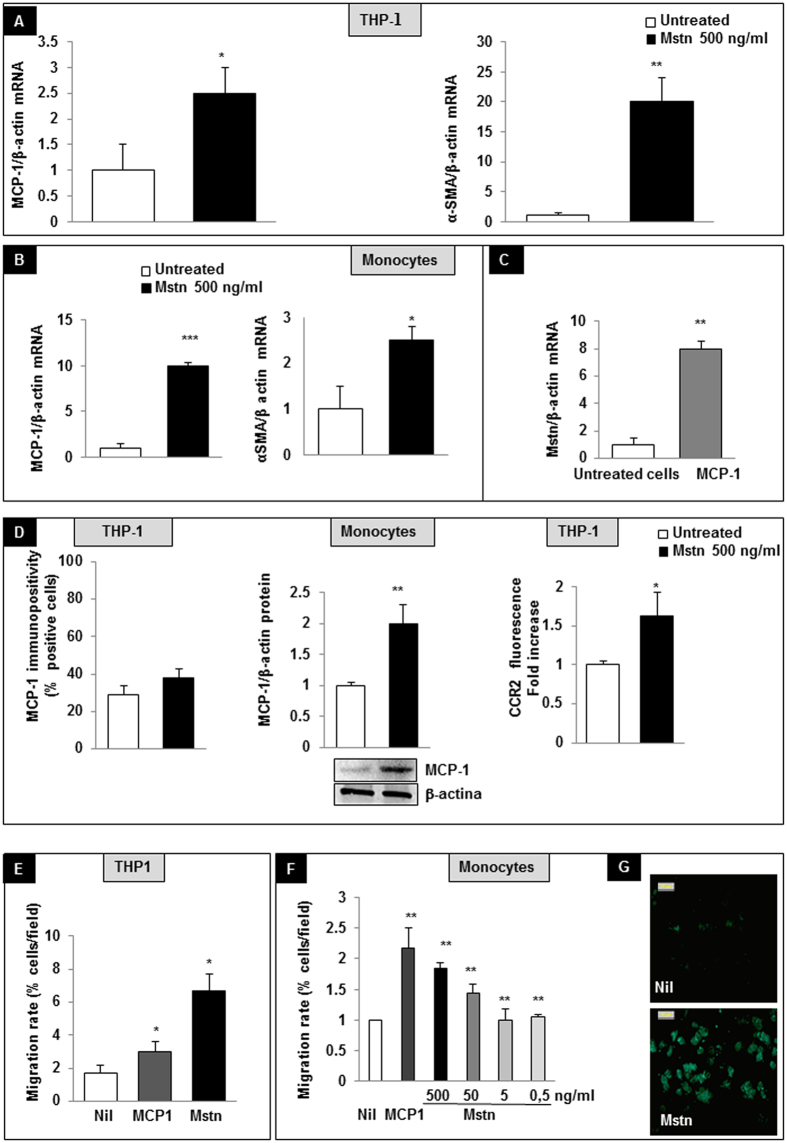
Effects of Myostatin (Mstn) on monocytes. (**A**) In THP-1 cell line, Mstn induces MCP-1 and α-smooth muscle actin (α-SMA) mRNAs. (**B**) In human freshly isolated monocytes, Mstn overexpressed MCP-1 and α-SMA mRNAs; (**C**) MCP-1 increased Mstn mRNA expression; mRNA levels were determined by real time PCR. All data were first normalized to β-actin mRNA and expressed as fold increase ± SEM versus untreated cells (**D**) MCP-1 and CCR2 protein expression; MCP-1 was evaluated in THP-1 cells by immunocytochemistry and in freshly isolated human monocytes by western blot after a 16 hour and one hour of Mstn treatment respectively. CCR2 membrane expression was evaluated on THP-1 cells after 8 hours of Mstn exposure. (**E**) Migration assay of THP-1 cells toward control medium, Mstn enriched medium (500 ng/ml), MCP-1 (as positive control). (**F**) Chemotaxis of freshly isolated monocytes, toward a Mstn-enriched medium at different concentrations. MCP-1 was used as positive control. (**G**) Mstn exposition caused F-actin polymerization distributed mainly in the cortical region, as demonstrated by AlexaFluor 488-Phalloidin staining (magnification 40x). *p < 0.05, **p < 0.01, ***p < 0.001 vs. untreated cells.

In THP-1 cells, Mstn increased MCP-1 and α-SMA mRNA expression (Fig. 6A) (2.4 ± 0.7 folds and 18.2 ± 4.3 folds versus untreated cells, respectively, after 16 a hour treatment; p < 0.01). A similar pattern was observed in human monocytes (9.8 ± 0.6 and 2.45 ± 0.73 folds vs. untreated cells respectively after a 16 hour treatment, p < 0.01. Fig. 6B). In turn, monocytes stimulated with MCP-1 displayed an increased Mstn mRNA expression (7.8 ± 0.45 fold increase after a 6 hour treatment, Fig. 6C) suggesting that Mstn participates in a feed forward inflammatory loop. When considering the protein expression, MCP-1 was slightly, not significantly increased in THP-1 cells after a 16 hour of Mstn treatment, while freshly isolated human monocytes displayed a two-fold increased MCP-1 expression within one hour of Mstn exposure. Accordingly, also CCR2 membrane expression was significantly upregulated following Mstn treatment (THP-1 cells, 8 hour exposure) (Fig. 6D).

#### Migratory rate and cytoskeletal organization

In THP-1 cells, Mstn (500 ng/ml) acted as a potent chemoattractant: when added to the lower well of a Boyden chamber, it caused a 5% and a 3.7% increase of the migratory rate compared to basal cell migration (Nil) and MCP-1 (10 ng/ml), the latter used as a positive control (Fig. 6E). Mstn also affected migration rate in a dose-dependent manner in human primary monocytes, reaching the highest rate at the concentration of 500 ng/ml, but being still effective at concentrations as low as 50 ng/ml, when compared to cell migration rate observed in untreated cells (Fig. 6F). A five hour Mstn exposition caused F-actin polymerization mainly in the cortical region, as demonstrated by AlexaFluor 488-Phalloidin staining (Fig. 6G), suggesting that Mstn modulates changes in the cytoskeletal organization for protrusive and contractile forces.

### Effects of Mstn on MAP kinase signaling pathways

**Figure 7:**
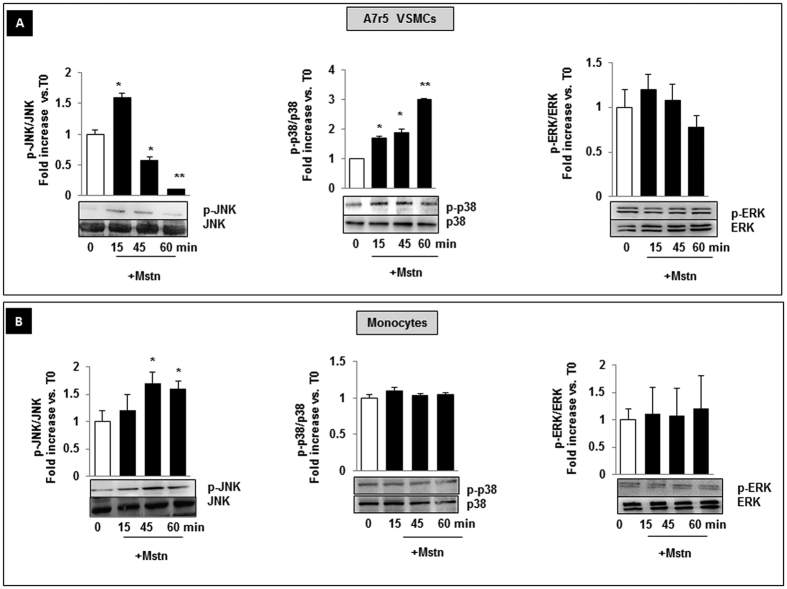
Effect of Myostatin (Mstn) on MAPKinase phosphorylation in A7r5 VSMCs and human freshly isolated monocytes. Cells were exposed to Mstn for 15, 45, 60 minutes and cell lysates were analyzed by western blot. Membranes were stripped and reprobed for non-phosphorylated MAPKs. Mstn induces phosphorylation of JNK and p38 in A7r5 cells (**A**), and of JNK in monocytes (**B**). All results represent mean ± SEM obtained from three independent experiments. *p < 0.05, **p < 0.01 vs T0. p-JNK = Phosphorylated JNK, p-ERK = Phosphorylated ERK, p-p38 = Phosphorylated p38.

Several *in vitro* studies revealed the involvement of MAP Kinases signaling in Mstn activity^30^. Therefore, we investigated what cellular pathway is activated by Mstn in A7r5 VSMCs and freshly isolated human monocytes. Both cells types exposed to Mstn for 15, 45, 60 minutes were compared to untreated cells (referred as T0). In A7r5 cells, phospho-JNK increased early (60% after 15 minutes, p < 0.05) and progressively declined within 60 minutes. Also p38 MAPK phosphorylation was upregulated after 15 min; this effect was maintained during the whole time course. No effect was observed on ERK activation (Fig. 7A). In monocytes, Mstn increased only JNK phosphorylation (of about 70 and 60% vs. T0, at 45 and 60 minutes respectively, p < 0.05) (Fig. 7B). Mstn did not change the ratio of phospho-p38 and phospho-ERK in respect to T0.

## Discussion

In this study, we provided elements to support the hypothesis that Mstn plays a role for the development of abdominal aortic atherosclerosis. To this aim, we examined the presence of Mstn and its association with specific markers of VSMC differentiation and of haematopoietic lineage in human samples of abdominal aortas. First, we observed that Mstn expression increases early during vascular wall activation, reaching the highest levels in the progressive atherosclerotic lesions.

Second, we provided evidence suggesting that Mstn, being mainly localized in the media-intima interface, is implicated in vascular remodeling of the abdominal aorta, and promotes phenotype-switching of VSMCs and of bloodstream-derived leucocytes (CD45+ cells) in correspondence of the lesion sites. We also established that Mstn affects proliferation and cytoskeletal architecture of VSMCs and the migratory rate and expression of proinflammatory cytokines of both VSMCs and monocytes. Finally, we identified in phospho-JNK a common pathway of activation elicited by Mstn both in A7r5 VSMCs and monocytes.

VSMCs and monocytes are recognized to be the major mediators for propagating and perpetuating inflammation throughout the vessel wall. Traditionally, the vascular damage was thought to proceed as an inside-out response to the subintimal modifications of low-density lipoprotein (LDL) cholesterol, which promotes recruitment of circulating leukocytes, SMC attraction from the media, and a defect in lesional macrophage egress. In addition to this, Maiellaro proposed a new component of outside-in inflammatory process responsible for the sustained activation of vascular cells and leukocytes during vascular remodeling, and observed that adventitial immune cells and other cells orchestrate an inflammatory process from the outer surface of the arterial tree^31^. Our finding of an early overexpression of Mstn in the tunica media, not necessarily adjacent to the atheroma formation, reinforce the hypothesis that a new component for the observed wall remodeling in the abdominal aorta might be constituted by Mstn-dependent VSMCs activation, which propagates from the media to the intima. In this perspective, Mstn should be further investigated for its role as a mechanical stress-induced signal in VSMCs. Indeed, in previous studies VSMCs have been already proven to sense variation of haemodynamic forces and wall stress distribution in a ROS-mediated pathway. The cell response to such cues may be reassumed as a change from a contractile to dysfunctional, synthetic phenotype, as indicated by the overexpression of the matricellular protein connective tissue growth factor (CTGF)^32^ and by the downregulation of markers of contractility, including Calponin 1 and Smoothelin^33^.

Smoothelin is a structural protein that actively participates to the cell contractile machinery and its loss leads to diminished vascular contractile capacity in mice^34^,^35^. We verified in our study that exposure of A7r5 VSMCs to Mstn caused a downregulation of their contractile machinery (decreased Smoothelin mRNA and α-SMA expression); Mstn also modulated stress fiber assembly and increased migratory rate in this cell line. Other features of VSMCs supplemented with Mstn are represented by a reduced proliferation rate, similarly to what was observed in other cell types^36^,^37^,^38^,^39^. This distinct VSMC phenotype has been described in humans and in vessels of other species as hallmarks of atheroma-prone phenotypes. Because plaque repair requires VSMCs to proliferate, defective proliferation and prolonged population doubling times have been viewed as features of advanced atherosclerotic lesions and of aged vessels in humans^40^. In this context, the finding that Mstn limits the proliferation rate of VSMCs may be viewed as a key factor for plaque stability and for the regulation of type and density of cell populations within atherosclerotic plaques.

VSMCs exposed to atherogenic lipids, cytokines and growth factors, and in aging recruit inflammatory cells, as demonstrated by the up-regulation of CCR2 and MCP-1^10^,^11^; in our experimental conditions, we observed that A7r5 VSMCs supplemented with Mstn upregulate MCP-1 and CCR2 expression. Overall, our data are supported by the recent findings of Janjanam and colleagues about the effects of MCP-1 in G-actin polymerization, F-actin stress fiber formation, and migration of human aortic SMCs^41^.

The finding of Mstn overexpression also in areas with bloodstream-derived leucocytes (CD45+ cells) support the notion that VSMC progenitor cells may originate in the vessel wall, in the circulation and in the bone marrow^42^. Simper *et al*. demonstrated that Mstn may influence SMC switching of hematopoietic mononuclear cells with angioblastic lineage that is distinct from endothelial outgrowth cells that differentiate into SMCs^43^. Yu *et al*. showed that cultured mouse marrow-derived SMC-like cells have an enhanced production of proinflammatory cytokine (MCP-1, granulocyte MCS factor, IL-6) in comparison to vessel wall VSMCs and exert a chemoattractant effect on monocyte-macrophages, which was reduced by neutralizing antibodies to MCP-1 and IL-6^44^. Thus, the long-standing and accepted paradigm that VSMC presence within lesions plays a beneficial role by promoting plaque repair and by protecting fibrous cap from rupture likely represents an oversimplification, because VSMC lineage may influence response to mitogens and other moieties present in aortic plaques^45^. It follows that promoting SMC survival in atherosclerotic plaques may have different biological effects: depending on SMC origin it might lead to an increased or decreased proatherogenic action.

We believe that our and previous findings indicate that Mstn expressed and released in the vessel wall represents a microenvironmental factor that can influence cellular processes and modulate VSMCs activity according to their lineage and/or their basal phenotypes.

When tested on monocytes, Mstn increased α-SMA expression and cell migratory rate, indicating a commitment toward an infiltrating phenotype^46^; in the same context, we also observed that Mstn and MCP-1 stimulate the reciprocal transcription rate in a feed-forward loop. These results, together with the evidences from the human peri-renal aortic specimens, indicate that Mstn is upregulated since the early stages of atheroma formation. Persistent Mstn expression is observed in later stages of vessel wall damage, where unabated inflammatory cell recruitment and dystrophic features of vascular damage are present^31^,^47^,^48^.

As mentioned before, heterogeneity for SMC phenotypes includes a wide combination of features, not confined to a mutually exclusive contractile or proliferative polarization. Increased proliferation and migratory rate of VSMCs are observed at early stages of atherosclerosis in adults with high levels of circulating lipids; enhanced profile of contractility is observed in patients with hypertension and Marfan-related vascular disease^9^; maladaptive calcification, impaired proteolytic activity, cell apoptosis and vessel wall thinning are displayed in AAA patients^49^. On this regard, Airhart and colleagues recently demonstrated that VSMCs that populate AAA have a unique elastolytic phenotype that is augmented in the presence of activated macrophages^50^.

More recently, the spectrum of VSMCs plasticity has been extended to a macrophage-like phenotype^51^.

To our knowledge, this study is the first to describe changes in the expression of Mstn in human aortae from subjects at different stage of atherosclerosic damage. Specifically, Mstn favors the switch of VSMCs toward a dystrophic phenotype and the accumulation of bloodstream derived monocytes expressing α-SMA, suggesting their commitment toward a fibroblast transition^52^.

Moreover, it offers insights into the potential mechanism by which Mstn induces vascular inflammation through JNK phosphorylation and the MCP-1-dependent crosstalk between VSMCs and recruited monocytes. Several *in vitro* studies performed in cell models revealed the involvement of MAPK signaling pathways in the effects of Mstn^30^,^53^ and JNK signal has long been recognized to be crucial for negative regulation of muscle growth^54^. More recently JNK activation in VSMCs has been shown to be essential in ADP-induced MCP-1 expression, promoting monocyte recruitment and vessel wall inflammation^14^.

In our setting, JNK phosphorylation emerged as a common mechanism of VSMCs and monocyte activation induced by Mstn; therefore, it may represent a terapeutical target to counteract the VSMC-monocyte interplay responsible for early atherogenesis development.

Dissecting new molecular mechanisms for atherosclerosis progression in the abdominal aorta would not only improve the understanding of the overall disease, but might also have profound clinical impact. Indeed, rupture of AAA, peripheral embolism from AAA or from damaged aortae, critical peripheral ischemia are among the most ominous consequence of accelerated abdominal aortic atherosclerosis and often constitute life-threatening conditions.

Future studies on Mstn may lay the ground for novel therapies to prevent late clinical complications by targeting proinflammatory activity of VSMCs and macrophages in the vessel wall.

## Materials and Methods

### Human specimens

This study was approved by the Ethics Committee of the University of Genova (Comitato Etico Regionale Liguria, Italy), and adhered to the Declaration of Helsinki Principles.

Patients and Tissue Sampling: Samples of non-aneurysmal abdominal aorta from age-matched, male subjects were obtained from the biobank of Leiden University Medical Center (LUMC) and classified according to the modified American Heart Association (AHA) classification as proposed in van Dijk *et al*.^26^. (Normal aorta-Norm, N = 8; adaptive intimal thickening-AIT, N = 12; Intimal xanthoma-IX, N = 12; pathological intimal thickening-PIT, N = 10; Early fibroatheroma-EFA, N = 9; late fibroatheroma-LFA, N = 5; Thin cap fibroatheroma-TCFA, N = 8; Plaque rupture-PR, N = 10; healed plaque rupture-HR, N = 11; fibrotic calcified plaque-FCP, N = 12).

Sample collection was performed in the LUMC in accordance with the guidelines of the local Ethics Committee and the and the code of conduct of the Dutch Federation of Biomedical Scientific Societies (http://www.federa.org/?s=1&m=82&p=0&v=4#827).

Tissue RNA was isolated using the Qiazol Lysis reagent (Qiagen Sciences, Maryland, USA) and isolated RNA was stored at −80 °C until use. Parallel formaldehyde fixed samples were used for immunohistochemical staining.

#### Histological preparation and immunohistochemical staining

Paraffin sections (5 μm) of 2% paraformaldehyde-fixed tissues were deparaffined, hydrated and subjected to antigen-retrieval (microwave oven treatment in 0.1 M sodium citrate). Staining was performed after quenching of endogenous peroxidase with 3% H_2_O_2_ in methanol. Slides were incubated with primary antibody overnight, followed by incubation with biotinylated antibody for 30 minutes. Each sample was analyzed for the detection of Mstn (mouse monoclonal ab, biorbyt, Cambridge, UK) and a labelled polymer HRP anti-mouse from Dako was used as secondary antibody. Staining with CD45 (Novocastra, Leica Microsystem, Milano Italy), α-SMA (Dako Italia s.r.l, Agilent Pathology Solution, Cernusco sul Naviglio, Italy) were completed with the appropriate secondary antibody using the streptavidin-peroxidase method, performed as previously described^55^. The expression of Mstn was examined by image analysis and expressed as positive areas. In order to evaluate the co-distribution of two different antigens in the same sample, a double immunohistochemistry procedure was carried out. First, one antibody was evidenced by streptavidin-peroxidase and the second by alkaline phosphatase-conjugated streptavidin (Vector Laboratories, CA, USA). The alkaline phosphatase substrate was Vector ^®^RED Substrate (Vector Laboratories).

#### mRNA Analysis

The RNA concentration and integrity of each sample were evaluated on a NanoDrop ND-1000 Spectrophotometer (NanoDrop Technologies Inc., Wilmington, DE, USA). 1 μg RNA was used for cDNA synthesis with RealMasterMix (5Prime, Eppendorf, Milan, Italy). Primers were obtained from Primerdesign (Southampton, United Kingdom) and Tibmolbiol (Genoa, Italy) and sequences are reported in Table 2. PCR amplification was carried out in a total volume of 10 μL, containing 1 μL cDNA solution, 5 μL SYBR solution Precision 2xqPCR MasterMix (Primerdesign), 0.5 μL each primer, 3.5 μL of nuclease-free water. Relative mRNA levels were calculated from cycle threshold (Ct) values using Vimentin as housekeeping gene.

### Cell cultures

#### Cell lines

Rat vascular smooth muscle cells (A7r5) and human monocytes (THP-1) were obtained from the European Collection of Cell Cultures (ECACC, UK). A7r5 cells were maintained in DMEM (Euroclone, Milan, Italy) culture medium supplemented with 10% heat inactivated fetal bovine serum (FBS), 2 mM Glutamine and 100 U penicillin-streptomycin; sub-confluent cell cultures were treated with Mstn (Peprotech, London UK) (500 ng/ml). THP-1 cells were maintained in RPMI (Euroclone) culture medium supplemented with 10% heat inactivated fetal bovine serum (FBS), 2 mM Glutamine and 100 U penicillin-streptomycin and treated with Mstn (500 ng/ml).

#### Human Monocyte Isolation

Human monocytes were isolated from buffy coats obtained from healthy volunteers, under a protocol approved by the local Ethics Committee. All donors provided a written, informed consent to the procedure and use of the cells. After centrifugation on a Ficoll-Hypaque density gradient, mononuclear cells were collected from the interface and washed with PBS. Monocytes were then purified from the upper interface of a hypotonic Percoll density gradient (1.129 g/mL). Purified monocytes were resuspended in RPMI 1640 medium supplemented with 1% (v/v) heat-inactivated fetal calf serum (FCS) and 500 ng/mL polymyxin B, as previously described^56^. At the end of purification, viability of monocytes was more than 98%, as determined by ethidium bromide-fluoresceinediacetate assay (from Sigma-Aldrich S.r.l., Milano, Italy). Monocyte purity was at least 90%, as assessed by flow cytometric analysis (staining with FITC-conjugated antihuman CD14 antibody (Ab), from BD Pharmingen, Franklin Lakes, NJ) and nonspecific esterase staining. Purified monocytes were treated with Mstn (500 ng/ml) or MCP-1 (10 ng/ml).

Treatments lasted for different time lags, on the basis of the experimental purposes, as reported in Results.

### Flow cytometry

#### Nuclear DNA content

Cells were permeabilized and nuclear DNA content was stained with a hypotonic solution (PI 50 μg/ml in Sodium Citrate/Triton X-100) at room temperature. The cell cycle analysis was performed using the ModFit LT 4.0 software (Verity Software House, Topsham, ME, USA).

#### Proliferation

Proliferation was evaluated by cell labelling with carboxyfluoresceinsuccinimidyl ester (CFDA-SE; Invitrogen, Milan, Italy). Data were analyzed with the Proliferation Wizard module of the ModFit LT 4.0 software (Verity Software House, Topsham, ME, USA) and the results were expressed as Proliferation Index.

#### CCR2 membrane expression

CCR2 was evaluated on THP-1 cells as median of cell fluorescence after staining with anti-human CCR2 Alexa Fluor 647 (clone 48607, Pharmingen).

Tests were performed using the Attune Acoustic Focusing Cytometer (Thermofisher Scientific).

### A7r5 cell dimension analysis

The area of A7r5 cells was evaluated after 24 and 48 hour exposure to Mstn (500 ng/ml) and compared with that of untreated cells. In brief, cells grown on chamber slides (EZ chamber slides, Millipore) were fixed with 2% paraformaldehyde, stained with Haematoxylin-Eosin and photographed with a Leica Microsystems microscope (GmbH Wetzlar, Germany) (100x magnification). Images were then analyzed using ImageJ software: the areas of randomly selected single cells were defined using the cursor and automatically estimated. The median values are calculated upon measurement of 150 cells/condition.

### F-Actin detection by fluorescence microscopy

A7r5 cells grown on EZ-chamber slides (Merckgroup) and freshly isolated monocytes were treated over night with Mstn (500 ng/ml). Cells were fixed with 2% paraformaldehyde, permeabilized with 0.05%Triton X 100, and stained with 5 U/ml Alexa-Fluor 488-conjugated phalloidin (A-12379 Alexa488 phalloidin; Molecular Probes). Cells were washed three times with PBS and viewed by fluorescence microscopy. Images were captured using the Leica Q500 MC image analysis system (Leica). Single images were digitized for image analysis at 256 gray levels. Imported data were quantitatively analyzed using Q500MC Software-Qwin (Leica). Constant optical threshold and filter combination were used.

### Migration assays

Migration assay was performed in a 48-well microchemotaxis chamber (Neuro Probe Inc., Gaithersburg, MD, USA) using a 8 μm pore size, polycarbonate polyvinylpyrrolidone-free filters (Millipore), by incubating the system loaded with cells at 37 °C. Test was performed on A7r5 cells, THP-1 cells and freshly isolated peripheral human monocytes.

Specifically, 5x10^4^A7r5 cells, suspended in 50 μL, untreated or incubated for 16 hours with Mstn (500 ng/ml), were washed with DMEM and placed in the upper wells. The lower wells of the chemotaxis chambers were filled with control medium DMEM alone (NIL), DMEM complete culture medium, or DMEM complete culture medium supplemented with Mstn (500 ng/ml). Cells were incubated for 6 hours.

One x10^5^ THP-1 cells suspended in 50 μL PBS were placed in the upper wells, while the lower compartment were filled with PBS (as negative control, NIL), MCP-1 (R&D Systems, 10 ng/ml in PBS) or Mstn (500 ng/ml in PBS). Incubation lasted for 3 hours.

One × 10^5^ human monocytes suspended in 50 μL of control medium (RPMI 1640+BSA 1%) loaded in the upper wells were let to migrate toward the lower wells filled with control medium alone (NIL), MCP-1 (10 ng/ml in control medium) or decreasing concentration of Mstn (500, 50, 5, 0.5 ng/ml in control medium). Cells were let to migrate for 3 hours.

After incubation, the filters were removed, fixed and stained with May Grunwald Giemsa (Carlo Erba, Italy). Each condition was performed in duplicate. Cells in five random fields were counted at oil-immersion 100x magnification (blind observer); the chemotaxis index was calculated as the number of cells migrated to the chemokine and divided by the number of cells migrated to the filter.

### cDNA reverse transcription and quantitative reverse transcription-PCR

Total RNA was extracted using TRIZOL reagent (Invitrogen) according to the manufacturer’s instructions. Total RNA (1 μg) was reverse-transcribed into cDNA by iSCRIPT RT Supermix (BioRad Laboratories, Inc. USA).

The gene expression of Mstn, Smoothelin, CCR2, and MCP-1 in A7r5 cells and of Mstn, a-SMA and MCP-1 in THP-1 cells and monocytes was quantified. The corresponding primers are reported in table 2 and PCR amplification was carried out as described above.

### Western blot analysis

Cells were lysed in cold buffer (20 mM HEPES, 150 mM NaCl, 10% [v/v] glycerol, 0.5% [v/v] NP-40, 1 mM EDTA, 2.5 mM DTT, 10 µg/L aprotinin, leupeptin, pepstatin A, 1 mM PMSF, and Na_3_VO_4_). Protein concentration was determined by using the Bicinchonic Protein assay kit (Merck Group, Vimodrone, Italy) and 10–20 μg were resolved on SDS-polyacrylamide gels and electro-transferred to a PVDF membrane (Merck Group). Blots were probed using anti CCL2/MCP-1 polyclonal antibody (Novus Biologicals, Space Import-Export s.r.l., Milan, Italy), anti phospho-ERK1(T202/Y204)/ERK2 (T185/Y187) polyclonal antibody (R&D Systems, Space Import-Export s.r.l., Milan, Italy), anti phospho-p38 MAP Kinase (T180/Y182) polyclonal antibody (R&D Systems), anti phospho-JNK (Thr 183/Tyr 185) (Santa Cruz Biotechnology), reprobed with β-actin or ERK, p38, JNK (Santa Cruz Biotechnology) and incubated in horseradish peroxidase secondary antibodies (Cell Signaling Technology). Immunoblots were developed with Immobilon Western chemiluminescent HRP substrate (Merckgroup). Band intensities were determined using Alliance system (Uvitec, Cambridge, UK).

### Immunocytochemistry

A7r5 grown on chamber slides to sub-confluence or THP-1 treated with Mstn (500 ng/ml) and spotted on slides were fixed in cold methanol and incubated with anti CCR2 polyclonal antibody (Novus Biologicals) or anti MCP-1 polyclonal antibody (Novus Biologicals). Immunostaining and image analysis were performed as described previously^56^.

#### Statistical analysis

Data are given as mean ± SEM. Statistical analysis was performed by Student’s t test or one-way Anova with Bonferroni’s post-test for multiple group comparison. Statistical significance was set at p < 0.05. All statistical analyses were performed using Graph Pad Prism version 5.00 for Windows, GraphPad Software, San Diego, California, USA. Spearman’s correlationwas used to demonstrate the relationship between Mstn expression and the type of lesions. A value of p < 0.05 was considered statistically significant.

## Additional Information

**How to cite this article**: Verzola, D. *et al*. Myostatin mediates abdominal aortic atherosclerosis progression by inducing vascular smooth muscle cell dysfunction and monocyte recruitment. *Sci. Rep.* **7**, 46362; doi: 10.1038/srep46362 (2017).

**Publisher's note:** Springer Nature remains neutral with regard to jurisdictional claims in published maps and institutional affiliations.

## Supplementary Material

Supplementary Figures

## Figures and Tables

**Table 1:** Summary of Myostatin (Mstn) immunopositivity within the different areas of the vessel wall in the different stages of plaque progression.

Lesion Type^6^	MEDIA (VSMCs)		NEOVESSELS		INTIMA/NEOINTIMA		LESION/FIBROTIC CAP		INFILTRATING CELLS		Total case number
	Posit.	N	Posit.	N	Posit.	N	Posit.	N	Posit.	N	
Norm	**0**	*6*	**na**		**0**	*8*	**na**		**na**		**N = 8**
	+/−	*2*									
AIT	+/−	*10*	**na**		**0**	*12*	**na**		**na**		**N = 12**
	+	*2*									
IX		*3*	**na**		**0**	*11*	**na**		**0**	*12*	**N = 12**
	+/++	*9*			+	*1*					
PIT	**0**	*2*	**0**	*9*	**0**	*10*	**na**		**0**	*10*	**N = 10**
	+/++	*7*	++	*1*							
	++	*1*									
EFA	**0**	*1*	**0**	*9*	**0**	*9*	**0**	*9*	**0**	*9*	**N = 9**
	+/++	*8*									
LFA	+/++	*3*	**0**	*3*	**0**	*1*	**0**	*3*	**0**	*3*	**N = 5**
	++	*2*	++	*2*	++	*4*	++	*2*	+	*2*	
TCFA	**0**	*1*	**0**	*5*	**0**	*1*	**0**	*4*	0	*5*	**N = 8**
	+/++	*4*	++	*3*	+/++	*7*	+/++	*4*	+	*2*	
	++	*3*							++	*1*	
PR	**0**	*1*	**0**	*1*	**0**	*10*	**0**	*10*	**0**	*1*	**N = 10**
	+	*9*	+ +	*9*					++	*9*	
HR	+	*11*	0	*9*	0	*8*	**0**	*5*	**0**	*9*	**N = 11**
			+++	*2*	+	*3*	+/++	*6*	+	*2*	
FCP	+/++	*12*	**0**	*6*	**0**	*5*	**0**	*7*	**0**	*6*	**N = 12**
			+	*6*	+	*7*	+/++	*5*	+	*6*	

Values in semi-quantitative units (0: absent; +/−: low positivity; +: medium positivity; ++: high positivity; +++: the highest positive sample, as determined by 2 blind observers) and the corresponding case numbers are reported. Na: not applicable to this stage of the disease process. Norm: Normal aorta; AIT: adaptive intimal thickening; IX: Intimal xanthoma; PIT: pathological intimal thickening; EFA: Early fibroatheroma; LFA: late fibroatheroma; TCFA: thin cap fibroatheroma; PR: Plaque rupture; HR: healed plaque rupture; FCP: fibrotic calcified plaque.

**Table 2:** Primer accession numbers and sequences.

Gene	Species	Accession Number	Primer Sequences (5′ = > 3′)	Prod. Length
Mstn	Human	NM_005259.2	Fwd: CCAAAGCTCCTCCACTCCGRev: GGGAGTACAGCAAGGGCC	782
α-SMA	Human	NM_001613	Fwd: AAGCACAGAGCAAAAGAGGAATRev: ATGTCGTCCCAGTTGGTGAT	76
CCL2	Human	NM_002982	Fwd: ACCGAGAGGCTGAGACTAACRev:AATGAAGGTGGCTGCTATGAG	122
β-Actin	Human	NM_001101.2	Fwd: CCTCGCCTTTGCCGATCCRev: CTCGTCGCCCACATAGGAAT	220
Vimentin	Human	XM_0115196 49.1	Fwd: GCAAAGATTCCACTTTGCGTRev: GAAATTGCAGGAGGAGATGC	122
Mstn	Rat	NM_019151.1	Fwd: GGCACTGGTATTTGGCAGAGTRev: AGGGATTCAGCCCATCTTCTC	160
Smoothelin	Rat	NM_0010130 49.2	Fwd: CCAGGAGTTCTACCGCTGTCRev: CAGTCCACCAGCATCCGTG	93
CCL2	Rat	NM_031530.1	Fwd: TGACAAATACTACAGCTTCTTTGGGRev: CAGTTAATGCCCCACTCACCT	123
CCR2	Rat	NM_021866.1	Fwd: CGAAACAGGGTGTGGAGGATTRev: ATCAGCATACTTGTGGCCCTT	136
GAPDH	Rat	NM_017008.4	Fwd: CTCTCTGCTCCTCCCTGTTCTRev: ATACGGCCAAATCCGTTCACA	106
