# Supplementary material for: Myostatin mediates abdominal aortic atherosclerosis progression by inducing vascular smooth muscle cell dysfunction and monocyte recruitment
Source: Sci Rep. 2017 Apr 13;7:46362. doi: 10.1038/srep46362 (PMC5390310; doi:10.1038/srep46362)
Supplement: Supplementary Figures [file srep46362-s1.pdf]

Supporting information

Myostatin mediates abdominal aortic atherosclerosis progression by inducing vascular smooth muscle cell dysfunction and monocyte recruitment

Author list: Verzola D[1], Milanesi S[1], Bertolotto M[2], Garibaldi S[3], Villaggio B[1], Brunelli C[3], Balbi M[3], Ameri P[3], Montecucco F[2,4], Palombo D[5], Ghigliotti G[3], Garibotto G[1], Lindeman JH[6], Barisione C[3]

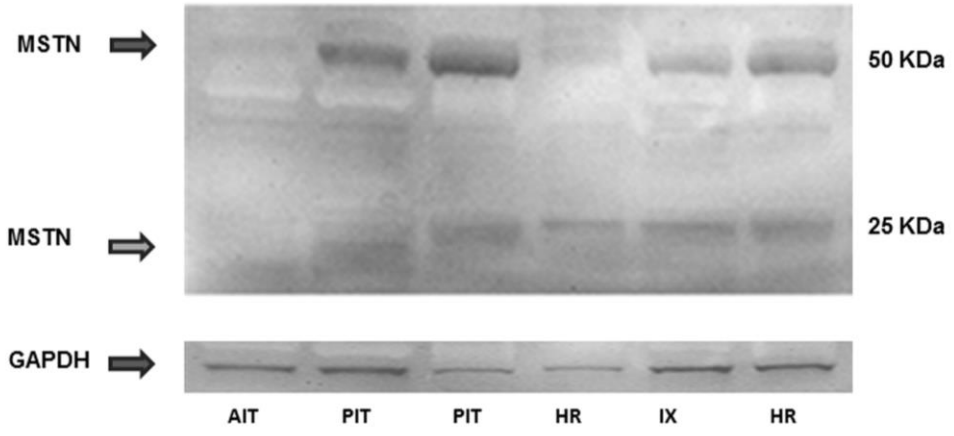

Figure S1 Western Blot for Mstn expression (recognized at approximately 50 KDa, as the precursor form and at 26 kDa as the secreted mature form) in abdominal aortic specimens at different stages of atherosclerotic lesion progression; as loading control GAPDH is used (37 KDa).

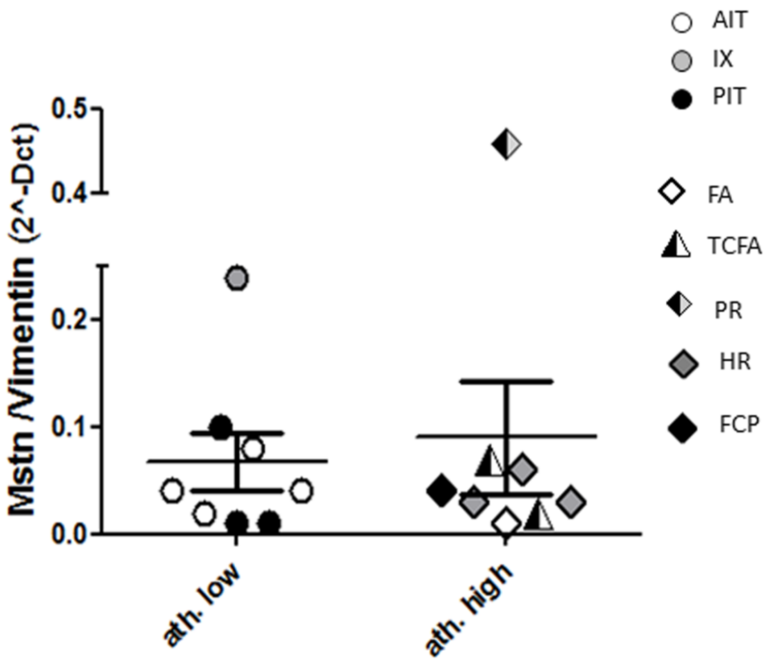

Figure S2 Comparison of Mstn gene expression in abdominal aortic specimens grouped as low or high stage of atherosclerotic lesion; Vimentin is used as housekeeping.
